# Perceptions of the Casemix system by clinicians after the first year of implementation in Hong Kong: a survey

**DOI:** 10.1186/1472-6963-11-S1-A21

**Published:** 2011-10-19

**Authors:** G Lam, SH Lee, D Yeung

**Affiliations:** 1 The Hong Kong Hospital Authority, Kowloon, Hong Kong

## Introduction

The Hong Kong Hospital Authority (HA) introduced a Pay-for-Performance (P4P) resource- allocation policy using a Casemix system in late 2008. Clinicians played a vital role in its implementation, especially with regard to the accuracy of clinical data. The purpose of this study was to:

1. Assess the short-term impact of Casemix-based funding as perceived by clinicians on clinical practice and quality of patient care after one year of implementation.

2. Examine any association between the characteristics of the clinicians (rank and specialty) and their perceived impact of the Casemix system on clinical service.

3. Identify the barriers encountered by clinicians on the effective implementation of this new policy.

## Methodology

A pilot quantitative study was done in March 2010 on a large, public general hospital in Hong Kong. All clinicians working in the hospital were recruited. A self-administered questionnaire, developed using recommendations from available literature, was used. Three aspects were looked at: the background characteristics of the clinicians, their perceptions about the impact of the Casemix system, and the clinicians’ knowledge of the Casemix system.

Five-point Likert scale, true-false, and open-ended questions were used. Analyses were performed to examine the relationship between the variables using Pearson’s chi-square test or Fisher’s exact test, where appropriate.

## Results

1. 520 questionnaires were sent out and the response rate was 17.3%.

2. More than 2/3 of the respondents did not perceive any change in their clinical practice or the quality of care, efficiency of work, or fairness of resource allocation.

3. More than 2/3 of the respondents agreed that there was improvement in clinical documentation, but at the expense of their time.

4. 60% of the respondents did not agree that the system induce gaming.

5. Participants’ knowledge of the Casemix system was generally poor, particularly among junior clinicians who, in addition, had a lower participation rate in the Casemix promulgation session conducted by the HA Casemix Office. The junior clinicians also expressed anxiety about having to carry out clinical documentation without being given clear guidance.

## Conclusions

After the first-year implementation of the P4P/Casemix policy in HA, clinicians did not perceive any negative impact on patient service, and they agreed that there was improvement in clinical documentation. The perceived lack of both knowledge and access to knowledge among the junior clinicians needs to be addressed. Lastly, a post-implementation survey was found to be useful in providing evidence to facilitate the formulation of communication strategies in the implementation of a corporate Casemix system.

